# Determinants of Postoperative Abdominal Wound Dehiscence among Patients Operated in a Tertiary Hospital

**DOI:** 10.4314/ejhs.v32i4.10

**Published:** 2022-07

**Authors:** Berhanetsehay Teklewold Teklemariam, Chaltu Fikru Biyana, Sahilu Assegid Asfaw

**Affiliations:** 1 Saint Paul's Hospital Millennium Medical College, Department of Surgery; 2 Jimma University, Department of Epidemiology

## Abstract

**Background:**

Abdominal wound dehiscence is one of the dreadful complications for surgeons in their daily activities.The Objective of this study was to identify determinants of abdominal would dehiscence among patients operated at Saint Paul hospital millennium medical college.

**Methods:**

A Matched case-control study to determine the predictors of abdominal wound dehiscence among operated patients at St. Paul's Hospital conducted. Multivariable logistic regression analysis done to calculate odds ratio and identify independent risk factors for abdominal wound dehiscnece.

**Result:**

A total of 68 cases and 68 controls were studied. Bivariable analysis revealed Preoperative sepsis, condition at admission, an indication of surgery, post-operative wound infection, and post-operative days of hospitalization as independent risk factor for abdominal wound dehiscence. Multivariable analysis proved the presence of preoperative sepsis and an indication of surgery as an independent risk factor. In-patient mortality was 9 (13.2%) in the dehiscence group. More than 90% of patients with dehiscence stayed more than ten days in hospital, but close to half of the controls stayed less than ten days(P<0.05)

**Conclusion:**

The presence of postoperative wound infection and an emergency surgical conditions were significant risk factors for an occurrence of postoperative abdominal wound dehiscence.

Focused follow-up of postoperative wound infection to identify signs of infection and meticulous implementation of perioperative infection prevention practices would save a lot more in a resource-limited setup.

## Introduction

Wound and its management are fundamental to the practice of surgery. Surgeons might face wounds either as primary pathology in case of trauma or they create wounds while trying to access other pathology in cases of elective surgeries. Therefore, surgical intervention will always result in wounds, and it is primarily; the surgeon's task to minimize the adverse effects of it; remove or repair damaged structures and harness the process of wound healing to restore function (1), but despite advances in wound management and surgical expertise wound management is still of a big concern to surgeons and their teams. Abdominal wound dehiscence (AWD) is a terminology used to explain separation of different layers of an abdominal wound before complete healing occurs. Other terms used interchangeably are acute laparotomy wound failure, burst abdomen, abdominal wound disruption, and evisceration. AWD usually occurs when a wound fails to achieve the required strength to withstand stresses placed upon it (2). It is one of the most feared postoperative complications for the surgeons and is of greatest regard because of the risk of burst abdomen, the need for immediate intervention, and the possibility of repeat dehiscence, surgical site infection, and mortality (3).

Its global incidence reported in several literature peaks from 0.4% to 3.5%, and mortalities associated with it are as high as 45% (4). In about 20–45% of cases, it becomes a significant risk factor associated with death during the perioperative period (2, 4). The high mortality, prolonged hospitalization, and increased incidence of reoperation, with their associated costs, emphasize the severity of the problem (5,6,7).

Several studies have identified many risk factors that are accountable for AWD, and a combination of these factors plays greater role than a single factor. Grossly, these factors could be classified as preoperative, perioperative, and postoperative, and good knowledge of these factors and early identification is mandatory and plays a significant role in reducting complications related to dehiscence (7, 8).

Access to surgical services is still a problem in developing countries (6), and several patients wait to get surgical services and among the factors that play a long waiting list of patients is the availability of beds and operation services (6,9). In a resource-limited setup like Ethiopia, it's vital to use hospital beds efficiently. One of the factor which influence bed availability is the presence of postoperative complications that prolongs hospital stay. This again increases the financial burden on the patient and affects proper and efficient utilization of limited beds in the institution. Therefore, This study aimed to determine predictors associated with abdominal wound dehiscence in those patients operated at Saint Paul's Hospital Millennium Medical College (SPHMMC).

## Materials and Methods

**Study Study area**: The study was done at SPHMMC, a tertiary teaching hospital in Addis Ababa, Ethiopia. St. Paul's hospital is one of the largest hospital in Addis Ababa and serves as a referral center for patients from Addis Ababa and all over the country. The Department of Surgery is one of the departments in the hospital and conducts both academic and clinical activities across different units.

**Study design and period**: A Matched case-control study was conducted from the 1st of July 2019 to the 1st of September 2019.

**Study population**: Surgical patients of all ages admitted to the Department of Surgery between the 1st of September 2014 and 30th of August 2019 and developed AWD postoperatively cases; and surgical patients of all ages admitted and operated on in the Department of Surgery between 1st of September 2014 and 30th of August 2019 and are matched to sex, age, and duration of admission to cases but did not develop AWD Post Operatively were controls.

**Sample size**: Sample size calculated using the formula for case control studies and 1 control for each case selected was used. Selected controls were matched for age, sex and similar period of admission. Seventy eight cases and 78 controls were needed in the study according to the calculation but the five-year review showed total of 71 patients to have AWD, and 3 patients had an incomplete chart and could not be included in the study. Therefore, a total of 68 patients with AWD and 68 controls were studied and analyzed.

**Inclusion criteria**: All patients of both sex who had developed partial and complete abdominal wound dehiscence after the first surgery, were included as a case in the study. Patients with inconclusive documents were excluded.

**Data collection and analysis**: A detailed case note of the patients' Socio-demography, and preoperative and postoperative clinical information was collected using a structured format. The data were analyzed using SPSS for windows version 23. Descriptive analysis were used for continuous and categorical variables. Texts, tables and charts, and cross tabs were used to display results. A comparison was made between cases and controls and the odds ratio, 95% confidence interval, and chi-square test were computed to assess the significance of the difference between groups. A p-value of < 0.05 is considered statistically significant. Multivariable analysis using stepwise logistic regression was done to study the relationship between variables.

Ethical clearance was obtained from the Institutional Review Board of SPHMMC. As this was a purely retrospective review of medical records, informed consent was waived by Saint Paul's Hospital Millennium Medical College IRB and therefore was not taken.

## Results

**Sociodemographic characteristics of the patients**: This study analyzed a total of 68 patients with dehiscence and 68 controls .The prevalence of AWD was calculated out of 2081 abdominal operations conducted within five years making the prevalence 3.2% (68/2081). There is a slight preponderance of male sex in the dehiscence group (3:1), and the commonest age group was age greater than 45 (41.2%) in dehisced patients. The mean age was 36 in both groups. Both age and sex have shown no significant association with dehiscence (Table 1).

**Table 1:** Socio-demographic characteristics of patients who had dehiscence and controls at surgical wards, SPHMMC from (1^st^ of September 2014 to 30^th^ of August, 2019)

Sociodemographic Variable	Cases No. (%)	Controls No. (%)	COR,95%CI	AOR,95%CI
Sex			1.74(0.8–3.6)	1.58(0.71–3.47)
**Male**	51 (75)	43 (63.2)		
Female	17 (25)	25 (36.8)		
Age group (in Years)			1.49(1.23–1.75)	1.24(0.83–1.65)
≤15	10 (14.7)	7 (10.3)	0.84(0.35–1.28)	0.74(0.33–1.20)
15–30	13 (19.1)	11 (16.2)	0.93(0.33–2.36)	0.51(0.23–2.28)
31–45	17 (25)	29 (42.6)	1.13(0.19–1.28)	1.02(0.16–1.21)
**>45**	28 (41.2)	21(30.9)	2.28(0.13–2.63)	2.03(0.10–2.43)

**Clinical characteristics of patients**: Forty-five percent of patients with dehiscence had some form of comorbidities 31(45.6%), and Anemia was the most common comorbidity identified among them 13 (42%). The majority of controls had no comorbidity 42 (61.8). Only 18(26.4%) patients with wound dehiscence had preoperative sepsis in contrast to the controls who had sepsis only in 7.4%; AOR with 95% CI: 4.55 (21.45–14.25). In the dehiscence group, 56 (82.4%) patients had surgery on an emergency basis, and only 12 (17.6%) had planned surgery; AOR with 95% CI: 2.62 (1.11–6.24). Close to one-third of the indication of the surgery 18(26.4%) was acute abdomen due to traumatic causes in dehisced groups, and only 3(4.4%) of patients in the control group had trauma as their cause; AOR with 95% CI: 6.07 (1.59–23.22). Vertical midline abdominal incision was the commonest type of incision done both on dehiscence and non-dehiscence groups 63(92.6%) and 65(95.6%) respectively; AOR with 95% CI: 0.41(0.07–2.29) (Table 2). Of 68 patients who had dehiscence, 51 (71.8%) had postoperative wound infection but in the control groups only 20 (28.2 %) had the wound infection; AOR with CI: 8.38(3.47–20.18) (Table 3).

**Table 2:** Clinical characteristics of patients who had dehiscence and controls at surgical wards, SPHMMC from (1^st^ of September 2014 to 30^th^ of August, 2019)

Preoperative Variables	Cases No. (%)	Controls No. (%)	COR,95%CI	P	AOR,95%CI	P
Co morbidity				0.345		0.178
Yes	31(45.6)	26(38.2)	1.35(0.70–2.76)		1.69(0.79–3.66)	
No	37(54.4)	42(61.8)	1		1	
Preoperative sepsis				0.005		0.005
Yes	18(26.4)	5(7.4)	4.53(1.5–13.6)		4.55(21.45–14.25)	
No	50(73.6)	63(92.6)	1		1	
Condition at admission				0.004		0.028
Emergency	56 (82.4)	41(60.3)	3..07(1.39–6.77)		2.62(1.11–6.24)	
Elective	12 (17.6)	27 (39.7)	1		1	
Type of Incision				0.47		0.309
Vertical	63 (92.6)	65(95.6)	0.58(0.13–2.5)		0.41(0.07–2.29)	
Transverse	5 (7.4)	3 (4.4)	1		1	
Indication				0.001		0.008
Traumatic	18(26.4)	3(4.4)	7.80(2.17–27.9)		6.07(1.59–23.22)	
Non traumatic	50(73.6)	65(95.6)	1		1	

**Table 3:** Management and outcome of patients who had dehiscence at surgical wards, SPHMMC from (1^st^ of September 2014 to 30^th^ of August, 2019) (n=68)

Post-operative variables	Cases No. (%)	Controls No. (%)	COR,95%CI	P	AOR,95%CI	P
Post-operative wound				0.001		0.001
Yes	51 (71.8)	20 (28.2)	7.2(3.3–15.3)		8.38(3.47–20.18)	
No	17 (26.2)	48 (73.8)	1		**1**	
Days of postoperative dehiscence						
0–5	20 (29.4)	0				
6–10	37 (54.4)					
11–15	10 (14.7)					
>15	1 (1.5)					
Days of hospitalization				0.001		0.001
<10 days	5(7.4)	33 (48.5)	11.88(4.25–33.18)		14.09(4.52–43.99)	
>10 days	63(92.6)	35(51.5)	1		**1**	
Outcome				0.085		0.901
Alive	59 (86.8)	62(91.1)	0.63(0.21–1.9)		0.92(0.24–3.49)	
Died	9 (13.2)	6(8.8)	1		**1**	

**Post-op management and outcome**: The commonest postoperative days in which dehiscence occurred were between 6–10 days which accounted for about 37 (54.4%) and rarely happened after the 15th postoperative day. Sixty-three patients (92.6%) from dehiscence groups were managed operatively, and tension closure was the commonest abdominal closure method used 24 (35.3%). The commonest operative finding during dehiscence surgery was an infection that accounted for 49 (77.9%), and loose and damaged sutures accounted for less than 10% of cases. In-hospital mortality was 9 (13.2%) in the dehiscence group and 6(8.8%) in controls. More than 90% of patients with dehiscence stayed more than 10 days in hospital, but close to half of the controls stayed less than ten days; AOR with 95%CI: 14.09(4.52–43.99) (Table 3).

**Factors associated with the occurrence of Abdominal Wound Dehiscence**: Sociodemographic and clinical characteristics were cross-tabulated with abdominal wound dehiscence as the primary outcome to identify independent risks. Preoperative sepsis, condition at admission, the indication of surgery, the presence of postoperative wound infection, and postoperative days of hospitalization were statistically significant upon bivariable analysis. When all these factors were regressed, except for preoperative sepsis and indication for first surgery all others proved not an independent risk factors on multivariable analysis (Table 4). Patients with pre-operative sepsis had more than two times higher risk of wound dehiscence when compared to controls but this was not statistically significant; AOR with 95% CI:2.65(0.61–11.56). Unplanned operations during emergency admissions had twice more increased risk of dehiscence than controls, and the presence of postoperative wound infection increases the risk of dehiscence ten times for cases when compared with controls. The Odds of patients with abdominal wound dehiscence staying in hospital for more than ten days is fourteen times higher than those without dehiscence; AOR with 95%CI 14.26(4.02–50.62). None of the factors were statistically significant when mortality analysed as an outcome.

**Table 4:** Multi variable analysis of risk factors related with wound dehiscence of patients a surgical wards, SPHMMC from (1^st^ of September 2014 to 30^th^ of August, 2019) (n=136)

Patient variables	Cases No. (%)	Controls No. (%)	AOR,95%CI	P
Preoperative sepsis				0.196
Yes	18(26.4)	5(7.4)	2.65(0.61–11.56)	
No	50(73.6)	63(92.6)	**1**	
Condition at admission				**0.021**
Emergency	56 (82.4)	41(60.3)	2.27(1.81–6.40)	
Elective	12 (17.6)	27 (39.7)	**1**	
Indication				0.076
Traumatic	18(26.4)	3(4.4)	1.86(0.15–2.56)	
Non traumatic	50(73.6)	65(95.6)	**1**	
Post-operative wound				**0.002**
Yes	51 (71.8)	20 (29.4)	10.66(3.98–28.52)	
No	17 (26.2)	48 (70.6)	**1**	
Days of hospitalization				**0.001**
< 10 days	5(7.4)	33 (48.5)	**1**	
> 10 days	63(92.6)	35(51.5)	14.26(4.02–50.62)	

## Discussion

Despite huge efforts in the field of surgery to reduce wound dehiscence postoperatively, there is still no change in the prevalence of abdominal wound dehiscence. Variety of factors are thought to be associated with its occurrence, and no two studies agree on the factors incriminated. This study was done to understand the magnitude of the problem and identify associated risk factors in tertiary hospitals in Addis Ababa.

The prevalence of wound dehiscence in our study is 3.2% (68/2081operations) which is in line with other reports showing ranges of prevalence between 0.2% and 6% (1–5, 13). The median age in our study was 36, and the commonly affected age group in dehisced patients was greater than 45 years(41%) in contrast to the mention of advanced age as one of the common risk factors listed in many literature (9–13). Reasons described in these literatures as a risk in advanced age is weakening of tissue repair as age gets older. The reason for the predominance of younger populations in our setup might be demographic change, and it might also be the changing pattern of disease in different settings globally. The similarity of the mean age between cases and controls shows the effect of matching during the selection. Our study showed patients with dehiscence are more commonly males with a little higher ratio of male to female (3:1) which is also consistent with other literature even though the finding was statistically insignificant (19). Male sex was also identified as a risk factor in almost several studies with the possible reason being that men have a higher abdominal wall tension, resulting in increased intra-abdominal pressure which in turn can lead to increased stress on the sutures resulting in the suture cutting through the fascia and muscles of the abdominal wall (24).

Our study showed that 45% of our patients had at least one form of comorbidity, and the commonest identified among those with comorbidity was Anemia (42%). Most literature mention anemia as a significant risk factor because of its role in tissue oxygenation. Anemia causes a decrease in tissue oxygenation, and subsequently, this leads to poor and delayed wound healing which later predisposes to dehiscence (2,25). Underlying malignancy, exposure to radiotherapy, and diabetes mellites were mentioned as significant risk factors for abdominal wound dehiscence according to Muhammed and his colleagues but our study did not show a significant association of such factors with dehiscence (35). Most of our patients were young and were not that predisposed to age-related comorbidities like diabetes and malignancy as described in other pieces of literature.

Emergency operations were also described as an important risk factor when compared to planned operations because of associated hemodynamic instability and the higher risk of operative site contamination because of inadequate preparation (4). In our study 82.4% (p<0.05) of patients in the dehiscence group had their surgeries during emergency hours, and the risk of dehiscence had been twice more than the controls, and this is also similarly indicated in other relevant literature consistent with our finding (2,5). Surgeons generally prefer vertical midline incisions because of their ease and associated excellent exposure, especially during emergency procedures. Higher rates of abdominal wound dehiscence were reported in vertical incisions as compared to transverse in several researches (9,10), and explanations given for this include the avascular nature of the vertical incision that eventually impairs appropriate wound healing postoperatively(10). Our study findings showed more than 90 % of patients with abdominal dehiscence had their operation done vertically, but there was no statistically significant difference between the two types of incisions among those with abdominal wound dehiscence and the controls. Wound dehiscence most commonly occurs from the fifth (5th) to the eighth (8th) postoperative day when the strength of the wound is at its weakest (16). Almost 90% of dehiscence occurs in less than 15 days postoperatively (17). In our study dehiscence had occurred between the first ten days in 84% of cases, and almost 99% of it had occurred in the first fifteen days postoperatively. This is similar to the reports of Van Ramshort and his associates which reported 90% of dehiscence to have occurred in less than 15 days (5). In this study presence of post-operative wound infection was identified as an independent risk factor among a few other factors and is found in 71.8% of the dehiscence group and less than 30% of control groups. The presence of postoperative wound infection increases the risk of dehiscence ten times for cases when compared with controls Relevance of wound infection as a risk for wound dehiscence is mentioned almost in every literature on a similar topic (5, 11 and 36). Smooth and orderly wound healing processes need to be free of the disruptive effect of different toxins and enzymes produced by bacteria in wound infection. During the earlier phases of wound healing collagen deposition on the wound site is very important and enzymatic degradation of collagen because of infection this time affects the strength of the wound adversely (20,21,24). Wound edges during earlier phases of healing should be supported by strong sutures and appropriate suturing techniques to hold edges together during abdominal closure. The importance of suture strength and techniques is also seen in our study by the fact that 9 % of findings postoperatively revealed loose and damaged sutures in the dehiscence group.

More than 60% of patients according to our study stayed between 20 to 30 days and this is also consistent with findings in other relevant pieces of the literature identified. Mazilu and his associates reported the average duration of a hospital to be 26 days (37) and Van Ramshore et al also reported a median duration of 36 days (5). Both studies also highlighted associated significantly higher mortality of patients with dehiscence when compared to controls, but our study has shown not that significant difference in mortality in both cases and controls respectively (13% versus 8.9%). The reason for this might be related to the difference in the distribution of age. In both studies, the mean age of patients was 65 and 70 years respectively and this is almost twice the mean age in our studies.

This study has several limitations and one of which is the failure to include several other variables that were very useful to learning associations of variables with dehiscence. Several kinds of research have shown an important association between surgical techniques, the expertise of the operating surgeon, and the type of suturing material used during abdominal closure. Identification of such variables from operative notes of patient charts will not be that difficult and future studies should consider including all factors that are incriminated as risk factors. The other important limitation of this study is overmatching of cases and controls which has prevented seeing the effect of age as a risk factor.

In conclusion, this case-control study has identified the association of different sociodemographic and clinical characteristics of surgical patients affecting surgical outcomes ranging from prolonged hospitalization to occurrence of dehiscence and finally mortality as an unwanted outcome. Based on the findings observed we can conclude that the presence of post-operative wound infection and emergency surgical conditions pose a significant risk of postoperative wound dehiscence and this, in turn, leads to increased morbidity and mortality as evidenced in our study by increased hospitalization and higher mortality.

Focused follow-up of post-operative wound infection to identify signs of infection and meticulous implementation of perioperative infection prevention practices would save a lot more in a resource-limited setup like ours. A coordinated effort of optimizing perioperative conditions to mitigate morbidities can be applied during the care of these patients.

**Ethical Approval**: Ethical clearance was obtained from institutional review board of Saint Paul's Hospital Millennium Medical College. As this was purely retrospective review on medical records, informed consent was waived by SPHMMC IRB and therefore was not taken.
